# A risk-scoring model for predicting late postoperative hemorrhage following pancreatoduodenectomy: development and external validation

**DOI:** 10.3389/fsurg.2026.1819332

**Published:** 2026-07-06

**Authors:** Shuai Xu, Qi Zhang, Liping Zang, Yuxiao Zhang, Jun Liu, Juan Zhang, Jianping Wang

**Affiliations:** 1 Department of Pancreatic Disease Diagnosis and Treatment Center, Shandong Provincial Hospital, Shandong University, Jinan, China; 2 Department of Liver Transplantation and Hepatobiliary Surgery, Shandong Provincial Hospital Affiliated to Shandong First Medical University, Jinan, China

**Keywords:** pancreatic fistula, pancreatoduodenectomy, post-pancreatectomy hemorrhage, prediction model, risk scoring model

## Abstract

**Background:**

Post-pancreatectomy hemorrhage (PPH) represents a life-threatening complication following pancreatoduodenectomy (PD). This study aimed to develop and externally validate a clinically applicable risk-scoring model to predict its occurrence.

**Methods:**

Patients who underwent curative-intent PD were included in the study. The risk-scoring model for predicting late PPH was developed in the training cohort, and the performance of the model was subsequently validated in an external validation cohort.

**Results:**

Of 405 eligible patients, 300 formed the training cohort and 105 the external validation cohort. Late PPH occurred in 7.7% and 7.6% of patients, respectively. Late PPH significantly impacted the long-term prognosis, with a median survival of 20.7 months compared to 35.2 months (*P* = 0.009). The most frequent site of hemorrhage was the common hepatic artery, accounting for 22.6% of cases. Multivariate logistic analysis identified body mass index (BMI), preoperative total bilirubin (TBIL), preoperative prothrombin time (PT), and clinically relevant postoperative pancreatic fistula (CR-POPF) as independent risk factors associated with late PPH. By integrating these four factors, the predictive model demonstrated concordance indices of 0.863 and 0.825 in the training and validation cohorts, respectively. The model's discriminative capacity was further assessed by categorizing the predicted probabilities of late PPH into two risk groups: low-risk (score ≤2) and high-risk (score >2).

**Conclusions:**

We developed and externally validated a simple risk-scoring model for late PPH after PD, using routinely available clinical variables to predict risk and stratify high-risk patients for targeted monitoring and prevention.

## Introduction

Pancreatoduodenectomy (PD) is a technically challenging abdominal procedure associated with significant morbidity and mortality rates (1, 2). Although PD has become relatively advanced in the treatment of carcinomas of the pancreatic head and periampullary region, yielding acceptable short-term outcomes (3, 4), postoperative complications such as delayed gastric emptying (DGE), postoperative pancreatic fistula (POPF), and gastrointestinal or intra-abdominal hemorrhage remain prevalent. Gastrointestinal or intra-abdominal hemorrhage occurs in 4% to 10% of all pancreatic resections and contributes to 11% to 38% of the overall mortality (5, 6). Although late post-pancreatectomy hemorrhage (PPH) is less common, it can result in severe consequences. Variability in pathophysiology and clinical presentation complicates the establishment of reliable diagnostic and therapeutic algorithms for managing late PPH. Consequently, the ability to clinically predict late PPH is of critical importance.

The International Study Group of Pancreatic Surgery (ISGPS) classifies PPH into early (onset ≤24 h post-operation) and late (>24 h post-operation) phases (5). Early PPH is primarily attributed to technical failures in achieving adequate hemostasis during the initial surgery or underlying perioperative coagulopathy. Conversely, late PPH typically results from postoperative complications, often manifesting several days to weeks after surgery. This is usually due to the erosion of a peripancreatic vessel secondary to a pancreatic fistula or intra-abdominal drains, ulceration at anastomotic sites, or the development of an arterial pseudoaneurysm (5, 7). Previous studies have identified numerous risk factors for PPH, including male sex, BMI ≥25 kg/m², diabetes mellitus, low geriatric nutritional risk index, antithrombotic use, postoperative pancreatic fistula (POPF), and hypertension (8–11). However, most existing models combine early and late PPH or focus on general PPH risk, and few provide a simple, bedside-calculable scoring system specifically for late PPH with rigorous external validation. Many existing tools rely on complex parameters or radiologic features that are not universally available in routine clinical practice (12, 13).

Against this background, this study used a multicenter cohort of patients undergoing PD to identify independent risk factors for late PPH and to develop and validate a clinically practical risk-scoring model for late PPH. The model was built in a training cohort and verified in an independent external validation cohort. By stratifying patients into low- and high-risk groups, this model may help clinicians implement targeted monitoring, early detection, and preventive measures to reduce the risk and improve outcomes of patients with late PPH.

## Methods

### Patient selection

The retrospective study was conducted from January 2017 to December 2020 at the Faculty of Liver Transplantation and Hepatobiliary Surgery, Shandong Provincial Hospital, affiliated with Shandong First Medical University. Patients who underwent curative-intent PD constituted the training cohort for the development of the risk score model. Patients who underwent PD at three other hospitals in China—The Jinan Central Hospital, The Linyi Cancer Hospital, and The Shanxian Central Hospital— formed an external validation cohort during the same period. This retrospective study on anonymized patient data was approved by Shandong Provincial Hospital (No.2022-178), and the requirement for additional board review and patient consent was waived. Anonymized data were collected at individual centers and subsequently collated and analyzed at Shandong Provincial Hospital.

### Patient variables and surgical procedures

The diagnostic criteria of tumor were based on typical preoperative serological and radiological features on intravenous contrast-enhanced computed tomography (CT), magnetic resonance imaging (MRI), or positron emission tomography-CT (PET-CT) and postoperative histopathological examinations. Patients were included if the tumor was considered to be resectable by a multidisciplinary team of clinicians based on preoperative examinations. All four participating centers routinely performed open pancreatoduodenectomy (OPD) and laparoscopic minimally invasive pancreatoduodenectomy (MIPD); however, none conducted robotic-assisted pancreatoduodenectomy (RPD) during the study period. All departments were high-volume pancreatic surgery centers with standardized perioperative management protocols and similar cumulative experience in open and laparoscopic PD. The detailed surgical procedures and extent of lymphadenectomy have been described in our previous studies (14, 15). During the surgical procedure, standardized electrosurgical devices were employed across all departments. With the exception of the ultrasonic scalpel length, no clinically or technically significant differences were observed between the MIPD and OPD approaches. Demographic data, pathology, and short- or long-term outcomes were analyzed. Postoperative complications were diagnosed and classified according to the ISGPS, the International Study Group of Liver Surgery (ISGLS) and the Clavien-Dindo classification (5, 16–19). Overall survival (OS) was calculated from the date of surgery to the date of death or the date of last follow-up. This study was censored on June 30, 2021.

### Inclusion and exclusion criteria

The inclusion criteria were patients (1) aged over 18 years; (2) with an American Society of Anesthesiologists (ASA) score of I-III; (3) underwent curative-intent PD; (4) benign low-grade malignant pancreatic tumors, resectable malignant pancreatic head tumors, ampullary tumors, or tumors at lower common bile duct (CBD). Resectable tumors with local vascular invasion were also enrolled. The exclusion criteria were patients (1) with unresectable vascular invasion or distant metastases; (2) a history of upper abdominal surgery; (3) incomplete data or follow-up lost.

### Statistical analysis

Continuous variables were reported as mean ± standard deviation (SD) or median [interquartile ranges (IQR)], and compared using the student's *t* test or Mann–Whitney *U*-test. Categorical data were reported as counts and percentages, and compared using the chi-squared test or the Fisher's exact test. X-tile software was used to identify optimal cutoff values for continuous variables based on outcome distribution, reducing investigator bias (20). Cutoffs were fixed before model building to avoid data-driven bias. Univariate and multivariate logistic regression methods were used to determine independent risk factors relating to risks of late PPH in patients in the training cohort. Linearity in the logit for continuous variables was confirmed using Box-Tidwell tests. No significant violations were observed. Variables with *P* < 0.05 in univariate logistic regression were entered into the multivariable model. A backward stepwise elimination method was used to identify independent risk factors, with retention criteria set at *P* < 0.05. Variance inflation factors (VIF) were calculated for all variables in the multivariable model. Regression coefficients (B) of the multivariate logistic regression model were rounded to the nearest unit (1.00 units) to obtain simple point numbers to facilitate bedside calculation of the scoring model. Validation of model performance was done in the external cohorts. Long-term survival curves were calculated using the Kaplan–Meier method and compared using the log-rank test. *P* values of less than 0.05 were considered as statistically significant with a two-tailed test. Data analyses were performed using SPSS (version 25.0, IBM, Armonk, New York, USA), R program (version 3.6.3, R Foundation for Statistical Computing, Vienna, Austria) and X-tile software (version 3.6.1, Yale University School of Medicine, New Haven, CT, USA). The main R packages including “survival”, “survminer”, “nomogramFormula”, “rms” and “ggplot2”.

## Results

### Clinical characteristics of patients

Patients who underwent curative-intent PD from four hospitals in China (*n* = 405) were divided into the training cohort (*n* = 300), and the external validation cohort (*n* = 105) (Supplementary Figure S1). A total of 263 patients underwent MIPD, with 196 patients (65.3%) in the training cohort and 67 patients (63.8%) in the validation cohort. Among the entire cohort of 405 patients undergoing PD, 87 patients (21.5%) received preoperative biliary drainage (PBD), mainly for cholangitis or severe jaundice. The incidences of late PPH in the two cohorts were 7.7% and 7.6%, respectively (*P* = 0.987). The baseline data for these patients was summarized in Table 1. There was no significant difference in baseline data between the training and external validation cohorts. The characteristics of the 31 patients who developed late PPH after PD are shown in Table 2. The median postoperative bleeding time was 9.0 and 10.5 days, respectively, for both cohorts. Extraluminal hemorrhages were more common than intraluminal hemorrhages, and branches of the common hepatic artery and stump of gastroduodenal artery (GDA) were more common. Arterial embolization was the most common means of hemostasis after PPH. Still, nearly a quarter of patients die as a direct result of PPH (Table 2).

**Table 1:** Clinicopathological characteristics of the two cohorts (*n* = 405).

Variables	Training Cohort	Validation Cohort	*P* value
	(*n* = 300)	(*n* = 105)	
Age (years)	62.0 (52.5, 68.0)	62.0 (54.3, 68.0)	0.654
Gender, male (%)	176 (58.7%)	59 (56.2%)	0.658
BMI (kg/m^2^)	23.3 (21.3, 25.2)	23.3 (20.8, 25.2)	0.506
History of alcohol use (year), (%)	26 (8.7%)	13 (12.4%)	0.267
History of smoking (year), (%)	58 (19.3%)	24 (22.9%)	0.439
Preoperative bile drainage, (%)	61 (20.3%)	26 (24.8%)	0.342
History of HBP, (%)	61 (20.3%)	18 (17.1%)	0.478
ASA grade, ≤ II (%)	255 (85.0%)	83 (79.0%)	0.158
Preoperative Hb (g/L)	124.0 (114.0, 135.0)	124.0 (108.5, 135.0)	0.970
Preoperative WBC (10^9^/L)	6.0 (5.0, 7.1)	5.8 (4.8, 7.5)	0.808
Preoperative PLT (10^9^/L)	230.0 (191.5, 286.0)	225.0 (184.0, 267.5)	0.466
Preoperative PT (S)	13.1 (12.6, 13.6)	13.1 (12.5, 13.7)	0.984
Preoperative APTT (S)	36.0 (34.0, 38.5)	36.1 (33.8, 39.1)	0.636
Preoperative INR	1.00 (0.95, 1.08)	1.01 (0.94, 1.07)	0.817
Preoperative FIB (g/L)	3.8 (3.1, 4.6)	3.8 (3.1, 4.8)	0.875
Preoperative BG (mmol/L)	5.2 (4.6, 6.1)	5.1 (4.8, 6.6)	0.504
Preoperative ALB (g/L)	38.6 (35.9, 40.7)	38.0 (35.7, 41.2)	0.601
Preoperative TBIL (mg/dL)	2.1 (0.7, 9.9)	1.8 (0.6, 8.3)	0.252
Surgical approach, MIPD (%)	196 (65.3%)	67 (63.8%)	0.778
Neoadjuvant chemotherapy, (%)	14 (4.7%)	5 (4.8%)	0.968
Operative time (min)	265.0 (240.0, 325.0)	265.0 (240.0, 315.0)	0.941
Estimated blood loss (ml)	190.0 (100.0, 235.0)	190.0 (100.0, 240)	0.748
Vascular resection, (%)	16 (5.3%)	6 (5.7%)	0.882
Resection margin, R0 (%)	271 (90.3%)	94 (89.5%)	0.811
Tumor location, (%)			
Pancreas	129 (43.0%)	38 (36.2%)	0.425
Bile duct	95 (31.7%)	35 (33.3%)	
Duodenum	76 (25.3%)	32 (30.5%)	
Pathology, malignant (%)	279 (93.0%)	95 (90.5%)	0.402
Largest tumor diameter (cm)	2.5 (1.5, 3.1)	2.3 (1.8, 3.0)	0.572
Peripheral tissue invasion, (%)	212 (70.7%)	75 (71.4%)	0.882
Tumor differentiation, (%)			
Benign and well	78 (26.0%)	30 (28.6%)	0.842
Moderate	132 (44.0%)	46 (43.8%)	
Poor	90 (30.0%)	29 (27.6%)	
CR-POPF, (%)^a^	37 (12.3%)	12 (11.4%)	0.807
Bile leak, (%)	20 (6.7%)	5 (4.8%)	0.485
Gastrointestinal fistula, (%)	5 (1.7%)	1 (1.0%)	0.958
Late PPH, (%)	23 (7.7%)	8 (7.6%)	0.987
Clavien-Dindo grade, ≥ III (%)	45 (15.0%)	15 (14.3%)	0.859
Postoperative LOS (d)	11.0 (9.0, 15.0)	12.0 (10.0,15.0)	0.761
90-day mortality, (%)	10 (3.3%)	4 (3.8%)	0.818

Number (Percentage)/Median (IQR); BMI, Body Mass Index; HBP, High blood pressure; ASA, American Society of Anesthesiologists; Hb, Hemoglobin; WBC, White Blood Cell; PLT, Platelet; PT, Prothrombin time; APTT, Activated partial thromboplastin time; INR, International Normalized Ratio; FIB, Fibrinogen; BG, Blood glucose; ALB, Albumin; TBIL, Total bilirubin; MIPD, minimally invasive pancreatoduodenectomy; CR-POPF, Clinically relevant postoperative pancreatic fistula; PPH, Post pancreatectomy hemorrhage; LOS, length of stay.

a Defined by the International Study Group on Pancreatic Surgery (2016).

**Table 2:** Characteristics of patients with late PPH in the two cohorts (*n* = 31).

Variables	Training Cohort (*n* = 23)	Validation Cohort (*n* = 8)	*P* value
Time of onset (days)	9.0 (6.0, 16.0)	10.5 (5.8, 13.8)	0.842
Tumor location, (%)			
Pancreas	8 (34.8%)	0 (0.0%)	0.076
Bile duct	12 (52.2%)	5 (62.5%)	
Duodenum	3 (13.0%)	3 (37.5%)	
Bleeding location, (%)^a^			
Intraluminal	3	1	
Gastrointestinal anastomosis	1 (33.3%)	0 (0.0%)	0.500
Bilioenteric anastomosis	2 (66.7%)	0 (0.0%)	
Jejunum stump	0 (0.0%)	1 (100.0%)	
Extraluminal	20	7	
Left gastric artery	3 (15.0%)	1 (14.3%)	>0.999
Common hepatic artery	5 (25.0%)	2 (28.6%)	
Proper hepatic artery	2 (10.0%)	0 (0.0%)	
Stump of gastroduodenal artery	4 (20.0%)	1 (14.3%)	
Branch of superior mesenteric artery	2 (10.0%)	1 (14.3%)	
Dorsal pancreatic artery	1 (5.0%)	0 (0.0%)	
Not defined	3 (15.0%)	2 (28.6%)	
PPH grade, (%)^a^			
B	10 (43.5%)	4 (50.0%)	>0.999
C	13 (56.5%)	4 (50.0%)	
Therapy, (%)			
Conservative treatment	1 (4.3%)	0 (0.0%)	0.737
Embolization	18 (78.3%)	6 (75.0%)	
Relaparotomy	4 (17.4%)	2 (25%)	
Mortality related to PPH, (%)	5 (21.7%)	2 (25.0%)	>0.999

a Defined by the International Study Group on Pancreatic Surgery (2007); PPH, post pancreatectomy hemorrhage.

**Figure 1:**
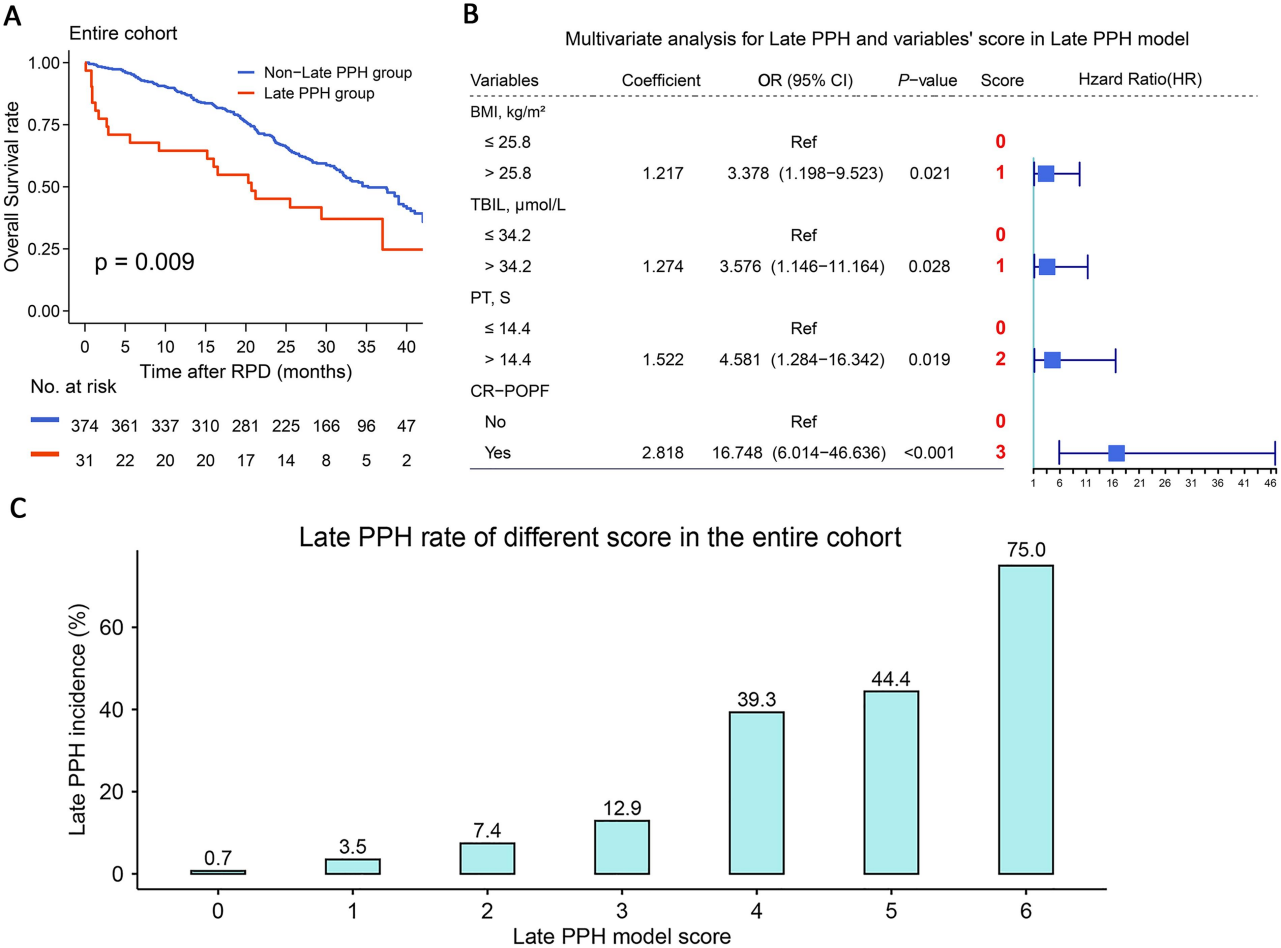
**(A)** the long-term survival outcomes of patients with or without late PPH after PD; **(B)** multivariate analysis for late PPH and variables’ score in the risk scoring model; **(C)** late PPH incidence of different score in the entire cohort. PD, pancreatoduodenectomy; PPH, post-pancreatectomy hemorrhage.

As shown in Table 3, compared with patients in the non-late PPH group, patients in the late PPH group had higher preoperative white blood cell (WBC) (6.9 vs. 6.0 * 10^9^/L, *P* = 0.026) and total bilirubin (TBIL) levels (7.7 vs. 2.0 mg/dL, *P* = 0.014), higher rates of bile duct tumors (54.8% vs. 30.2%, *P* = 0.018) and clinically relevant postoperative pancreatic fistula (CR-POPF) (58.1% vs. 8.3%, *P* < 0.001), as well as longer postoperative hospital stays (17.0 vs. 11.0, *P* < 0.001). In addition, the long-term prognosis of patients without postoperative bleeding was significantly better than that of patients with postoperative bleeding (35.2 vs. 20.7 months, *P* = 0.009) (Figure 1A).

**Table 3:** Patient characteristics of late PPH group and Non-late PPH group (*n* = 405).

Variables	Late PPH group	Non-Late PPH group	*P* value
	(*n* = 31)	(*n* = 374)	
Age (years)	62.0 (58.0, 69.0)	62.0 (52.8, 68.0)	0.189
Gender, male (%)	23 (74.2%)	212 (56.7%)	0.058
BMI (kg/m^2^)	23.4 (21.8, 26.4)	23.3 (21.1, 25.1)	0.225
History of alcohol use (year), (%)	4 (12.9%)	35 (9.4%)	0.744
History of smoking (year), (%)	10 (32.3%)	72 (19.3%)	0.083
Preoperative bile drainage, (%)	7 (22.6%)	80 (21.4%)	0.877
History of HBP, (%)	8 (25.8%)	71 (19.0%)	0.357
ASA grade, ≤ II (%)	23 (74.2%)	315 (84.2%)	0.149
Preoperative Hb (g/L)	129.0 (119.0, 141.0)	123.0 (112.0, 135.0)	0.051
Preoperative WBC (10^9^/L)	6.9 (5.3, 8.5)	6.0 (5.0, 7.3)	**0**.**026**
Preoperative PLT (10^9^/L)	242.0 (166.0, 289.0)	229.0 (189.8, 282.3)	0.795
Preoperative PT (S)	12.9 (12.3, 14.5)	13.1 (12.6, 13.7)	0.992
Preoperative APTT (S)	36.1 (34.6, 39.8)	36.0 (33.9, 38.9)	0.458
Preoperative INR	1.02 (0.92, 1.14)	1.01 (0.96, 1.07)	0.683
Preoperative FIB (g/L)	3.8 (3.2, 5.8)	3.8 (3.1, 4.6)	0.124
Preoperative BG (mmol/L)	5.4 (4.9, 6.6)	5.1 (4.7, 6.2)	0.159
Preoperative ALB (g/L)	37.9 (36.3, 40.1)	38.3 (35.7, 40.8)	0.639
Preoperative TBIL (mg/dL)	7.7 (1.5, 13.1)	2.0 (0.6, 8.9)	**0**.**014**
Surgical approach, MIPD (%)	18 (58.1%)	245 (65.5)	0.404
Neoadjuvant chemotherapy, (%)	2 (6.5%)	17 (4.5%)	0.630
Operative time (min)	290.0 (243.0, 340.0)	265.0 (240.0, 320.0)	0.193
Estimated blood loss (ml)	200.0 (150.0, 240.0)	185.0 (100.0, 235.0)	0.275
Vascular resection, (%)	1 (3.2%)	21 (5.6%)	0.573
Resection margin, R0 (%)	28 (90.3%)	337 (90.1%)	0.969
Tumor location, (%)			
Pancreas	8 (25.8%)	159 (42.5%)	**0**.**018**
Bile duct	17 (54.8%)	113 (30.2%)	
Duodenum	6 (19.4%)	102 (27.3%)	
Pathology, malignant (%)	28 (90.3%)	346 (92.5%)	0.929
Largest tumor diameter (cm)	2.0 (1.5, 2.7)	2.5 (1.8, 3.3)	0.068
Peripheral tissue invasion, (%)	24 (77.4%)	263 (70.3%)	0.403
Tumor differentiation, (%)			
Benign and well	7 (22.6%)	101 (27.0%)	0.854
Moderate	14 (45.2%)	164 (43.9%)	
Poor	10 (32.2%)	109 (29.1%)	
CR-POPF, (%)^a^	18 (58.1%)	31 (8.3%)	**<0**.**001**
Bile leak, (%)	2 (6.5%)	23 (6.1%)	>0.999
Gastrointestinal fistula, (%)	1 (3.2%)	5 (1.3%)	0.382
Postoperative LOS (d)	17.0 (13.0, 31.0)	11.0 (9.0, 14.7)	**<0**.**001**
90-day mortality, (%)	7 (22.6%)	7 (1.9%)	**<0**.**001**
Median survival time (months)	20.7 (10.5–30.9)	35.2 (32.1–38.3)	**0**.**009**

Number (Percentage)/Median (IQR); PPH, Post pancreatectomy hemorrhage; BMI, Body Mass Index; HBP, High blood pressure; ASA grade, American Society of Anesthesiologists physical status classification; Hb, Hemoglobin; WBC, White Blood Cell; PLT, Platelet; PT, Prothrombin time; APTT, Activated partial thromboplastin time; INR, International Normalized Ratio; FIB, Fibrinogen; BG, Blood glucose; ALB, Albumin; TBIL, Total bilirubin; MIPD, minimally invasive pancreatoduodenectomy; CR-POPF, Clinically relevant postoperative pancreatic fistula; LOS, length of stay.

Bold text hinted that these variables were statistically significant.

a Defined by the International Study Group on Pancreatic Surgery (2016).

### Development of a risk scoring model for late PPH prediction in the training cohort

For the convenience of clinical practice, the continuous variables were divided into two grades by X-tile. The 25.8 kg/m^2^, 2.0 mg/dL, and 14.4 S were the optimal cut-off values of preoperative BMI, TBIL and prothrombin time (PT), respectively. Multivariate logistic regression analysis showed that BMI [>25.8 vs. ≤25.8 kg/m^2^, odds ratio (OR) 3.378; 95% confidence interval (CI) 1.198–9.523; *P* = 0.021], TBIL (>2.0 vs. ≤2.0 mg/dL, OR 3.576; 95% CI 1.146–11.164; *P* = 0.028), PT (>14.4 vs. ≤14.4 S, OR 4.581; 95% CI 1.284–16.342; *P* = 0.019), CR-POPF (yes vs., no, OR 16.748; 95%CI 6.014–46.636; *P* < 0.001) were independent risk factors for late PPH. Detailed univariate and multivariate logistic regression analysis results are shown in Supplementary Table S1 and Figure 1B.

The scoring model was then developed to predict late PPH risks for patients after PD based on the regression coefficients (B) of each independent risk factor in the logistic regression model (Figure 1B). Points were assigned as follows: BMI >25.8 kg/m^2^: 1 point; TBIL >2.0 mg/dL: 1 point; PT >14.4 s: 2 points; Presence of CR-POPF: 3 points. According to the scoring model, the incidence of late PPH increased significantly as the patient's score increased (Figure 1C and Supplementary Table S2).

### Performance of the risk scoring model for late PPH prediction in the training and external validation cohorts

**Figure 2:**
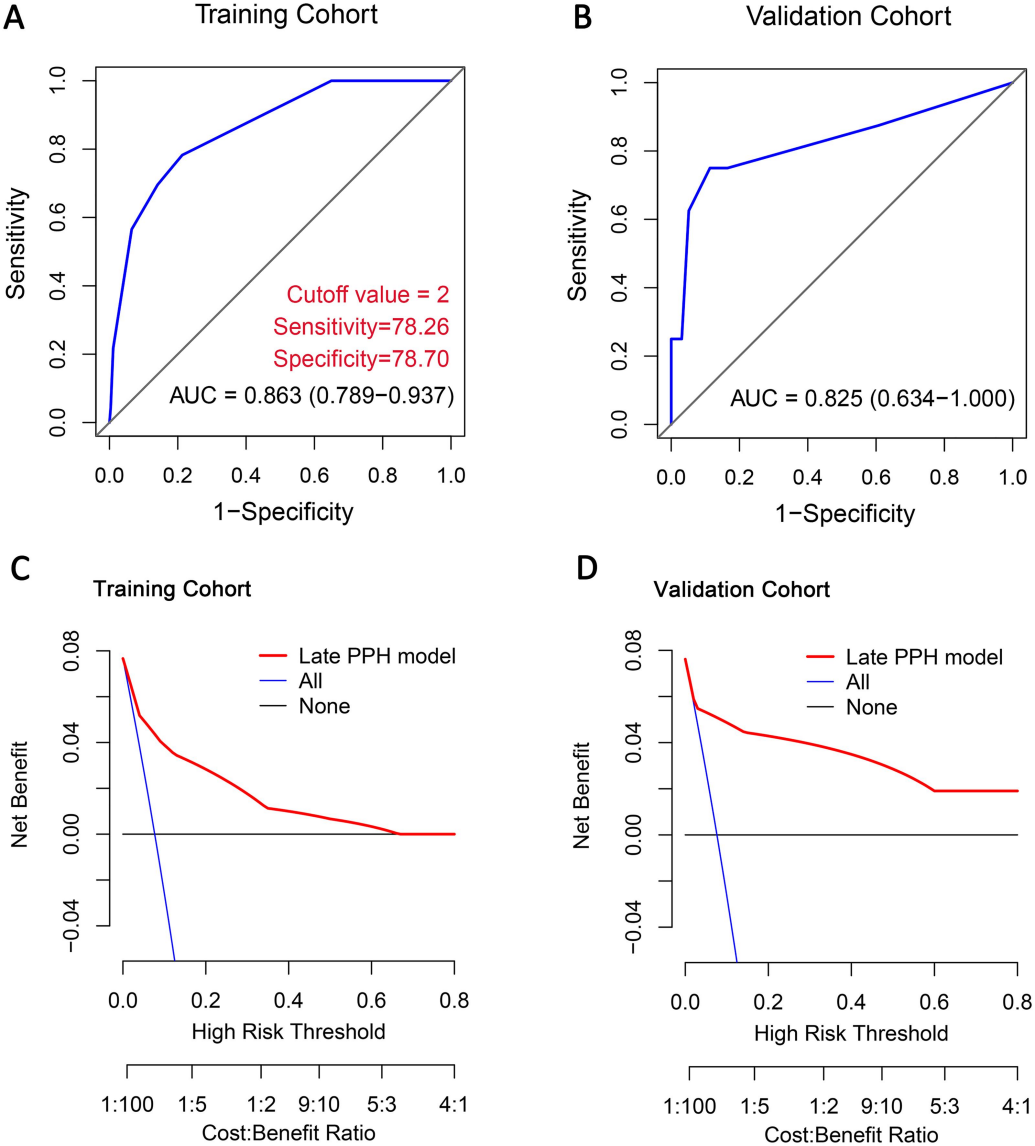
**(A,B)** receiver operating characteristic (ROC) curve analysis for the risk scoring model in the training and validation cohorts; **(C,D)** decision curve analysis (DCA) for the risk scoring model in the training and validation cohorts.

The area under the receiver operating characteristic (ROC) curve (AUC) of the model were 0.863 (95% CI 0.789–0.937), and 0.825 (95% CI 0.634–1.000) for prediction of late PPH risks after PD in the training and external validation cohorts, respectively (Figures 2A,B). In the subset of patients without CR-POPF, the scoring model retained good discriminative ability with an AUC of 0.762 (95% CI: 0.668–0.855) in the training cohort and 0.715 (95% CI: 0.526–0.904) in the external validation cohort.

Decision curve analysis (DCA) converts complex mathematical models into simple and easy-to-understand graphics for display (21). As shown in Figures 2C,D, within the threshold range of 0.1–0.8, the net benefit of the scoring model in predicting late PPH was significantly better than that of complete intervention or no intervention in the training cohort and external validation cohort.

### Discrimination of the risk scoring model for late PPH prediction in the training and external validation cohorts

**Figure 3:**
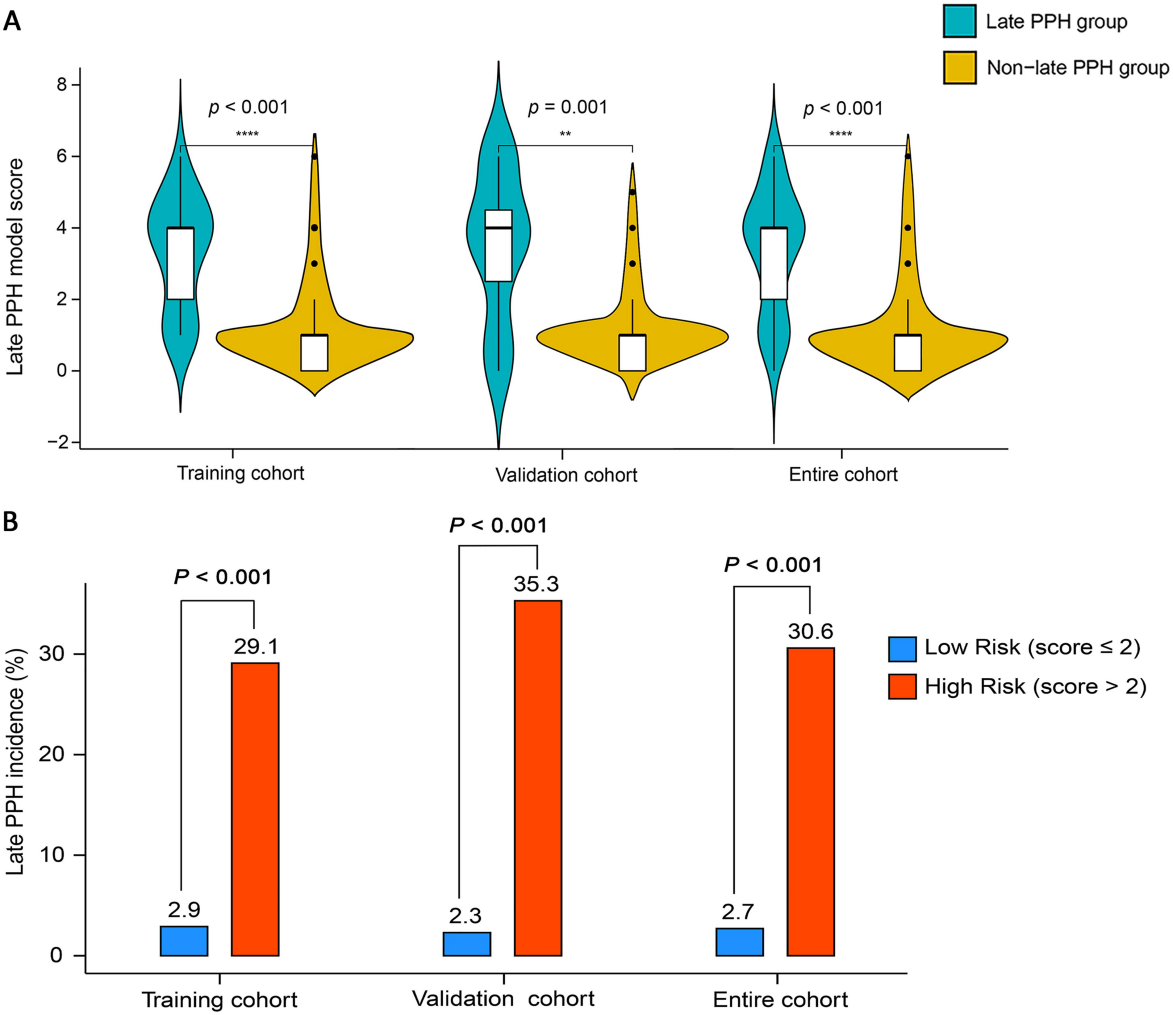
**(A)** violin plot shows the distribution of the model scores in patients with or without late PPH. **(B)** Incidence of late PPH in different risk groups. PPH, post-pancreatectomy hemorrhage.

As shown in Figure 3A, the scores of patients who experienced late PPH were also significantly higher than those of patients who did not experience late PPH in both the training and validation cohorts (*P* < 0.001). The ideal cut-off value for a patient's score was 2 points (Figure 2A). The discrimination ability of the model was further evaluated by dividing the predicted probabilities of late PPH risks into two risk groups according to the different scores (Low-risk group ≤2 points; High-risk group >2 points). The incidences of late PPH in the training cohort for the low, and high-risk groups were 2.9%, and 29.1%, respectively (Supplementary Table S3, *P* < 0.001). The scoring model performed equally well in the external validation cohort with significant differences in the rates of late PPH after PD (2.3% vs. 35.3%, *P* < 0.001) (Figure 3B and Supplementary Table S3).

## Discussion

PPH remains a life-threatening complication after PD, and late PPH in particular is associated with high mortality and poor long-term survival (7, 22). Although many studies have investigated risk factors and predictive models for PPH in large-scale and high-volume settings, most focus on overall PPH rather than late-onset hemorrhage, and few provide a simple, clinically applicable scoring system with external validation (12, 23, 24). The present study developed and validated a practical risk-scoring model for late PPH using routinely available clinical variables, with clear risk stratification for clinical use.

PD is widely used for benign and malignant tumors of the pancreatic head and periampullary region. Despite improvements in surgical techniques, postoperative complications, especially late PPH, still lead to adverse outcomes (11, 25, 26). Late PPH presenting over 24 h after operation can result from vessel erosion due to anastomotic leakage, intra-abdominal infection, vascular injury during surgery or postoperative pseudoaneurysm formation (27). However, diagnosis, treatment, and prognosis of late PPH are still imprecise, and it is controversial whether late PPH can be predicted. In this multicenter study of 405 patients, we constructed and verified a risk-stratification scoring model for late PPH with favorable performance and clinical feasibility.

The incidence of late PPH in our study was 7.6%–7.7%, consistent with previous reports ranging from 3% to 16% (28, 29). Multiple risk factors for PPH have been reported, including male sex, high BMI, diabetes, coagulopathy, malnutrition, CR-POPF, hyperbilirubinemia, and hypertension (12, 13, 23, 24). However, few models integrate these factors to predict late PPH specifically. Our study confirmed BMI, preoperative TBIL, preoperative PT, and CR-POPF as independent risk factors. These indicators are easily accessible in routine clinical practice, supporting the model's clinical utility.

Based on these results, alleviating jaundice and suspending antithrombotic therapy prior to surgery may reduce the risk of late PPH. Of note, patients with mild hyperbilirubinemia (TBIL 2.0–14.6 mg/dL) without infectious or systemic indications did not undergo routine PBD, consistent with standard clinical practice to avoid drainage-related complications (30). Preoperative TBIL was retained as an independent predictor in our scoring model not to advocate routine drainage, but to reflect the pathophysiological link between cholestasis, coagulopathy, and late PPH. Our results support guideline-aligned, selective PBD rather than universal drainage for patients with even mild bilirubin elevation. Further studies on these topics should be conducted (11, 31).

Previous studies have shown late PPH from a pseudoaneurysm of GDA stump usually resulted from POPF (25), and the median onset time of PPH was 5 to 13 days (7, 29). Our study shows similar results and highlights the importance of looking for PPH in patients with POPF after PD, especially in patients predicted by the scoring model to be at high risk of PPH. In the present study, we observed that patients with late PPH had significantly longer postoperative hospital stays than those without late PPH (17.0 vs. 11.0 days, *P* < 0.001). This finding is consistent with the reasoning: CR-POPF and other perioperative complications not only directly trigger vascular erosion and late PPH but also extend the length of hospital stay. Of note, we recognize that CR-POPF carries a relatively high weight in the final scoring model, which is attributable to its strong independent predictive value in multivariate logistic regression rather than arbitrary assignment. To confirm that the model provides incremental value beyond CR-POPF alone, we performed the subset of patients without CR-POPF, the scoring model retained discriminative ability with an AUC of 0.762 in the training cohort and 0.715 in the external validation cohort, indicating that BMI, preoperative TBIL, and PT still provided independent predictive information for late PPH in the absence of pancreatic fistula. These findings demonstrate that our model is not merely a proxy for CR-POPF. The integrated score improves risk stratification by combining preoperative factors with a postoperative complication, allowing early risk estimation before CR-POPF is diagnosed and more precise stratification afterward. This dual-phase predictive capacity enhances clinical utility compared with using CR-POPF alone.

Some previous studies have used radiologic features to predict late PPH, such as GDA stump diameter and intra-abdominal fluid collection (13). In contrast, our model relies solely on routinely available clinical variables—excluding imaging findings—to enhance practical applicability. It demonstrated robust and consistent discriminative performance in both the training (AUC = 0.863) and external validation (AUC = 0.825) cohorts. The minimal AUC decrement of 0.038 suggests negligible overfitting. DCA further confirmed a clinically meaningful net benefit across a broad range of threshold probabilities. A cutoff of 2 points clearly stratified low-risk and high-risk patients, enabling personalized monitoring and preventive interventions. Such a stratification would allow future studies to be conducted on high risks patients using close follow-up to detect early sentinel bleeding, and on preventive hemostasis therapies (32).

Previous studies (7, 32, 33) showed that conservative treatment or transcatheter arterial embolization should be the first-line treatment for late PPH, and surgical treatment should be offered after failure of interventional radiology or in patients who are hemodynamically unstable. Such a management approach using conservative, interventional, and surgical therapies was adopted in our study of patients with late PPH. Finally, as patients after PD with late PPH in our study had significantly worse long-term survival outcomes than those patients without late PPH irrespective of the nature of tumor and its location, risk prediction and preventive measures for late PPH after PD before bleeding happens are important to improve outcomes of patients undergoing PD.

There are several limitations to this study. First, although we utilized an external validation cohort to improve the reliability of the scoring model, the retrospective design and inherent patient selection may still introduce potential bias. Second, minor variations in surgical techniques and perioperative management protocols for PD across the participating centers may represent an additional source of heterogeneity. Third, the potential impact of the surgeons' learning curves on postoperative outcomes following PD was not evaluated in this analysis. Fourth, our model was developed in a cohort of patients undergoing upfront resection without neoadjuvant therapy, and that the predictive value of TBIL in patients receiving neoadjuvant treatment and preoperative biliary drainage needs further validation in future studies. Finally, because all participants were enrolled from Chinese medical centers, the generalizability of our model to other populations, especially Western cohorts, remains to be confirmed. Future multi-ethnic, international validation studies are warranted to evaluate the broader clinical applicability of this model.

## Conclusions

In conclusion, this multicenter study identified key risk factors for late PPH after PD and developed a simple, externally validated risk-scoring model for late PPH. The model uses routine clinical parameters to accurately identify high-risk patients and support targeted postoperative surveillance and preventive strategies, thereby helping to improve clinical outcomes.

## Data Availability

The datasets presented in this article are not readily available because limitation. Requests to access the datasets should be directed to Shuai Xu at: xs171111@163.com.
