# Supplementary material for: A risk-scoring model for predicting late postoperative hemorrhage following pancreatoduodenectomy: development and external validation
Source: Front Surg. 2026 Jul 6;13:1819332. doi: 10.3389/fsurg.2026.1819332 (PMC13403329; doi:10.3389/fsurg.2026.1819332)
Supplement: Supplementary file 1 [file Supplementaryfile1.docx]

# Supplementary Figure 1. Flowchart of our study


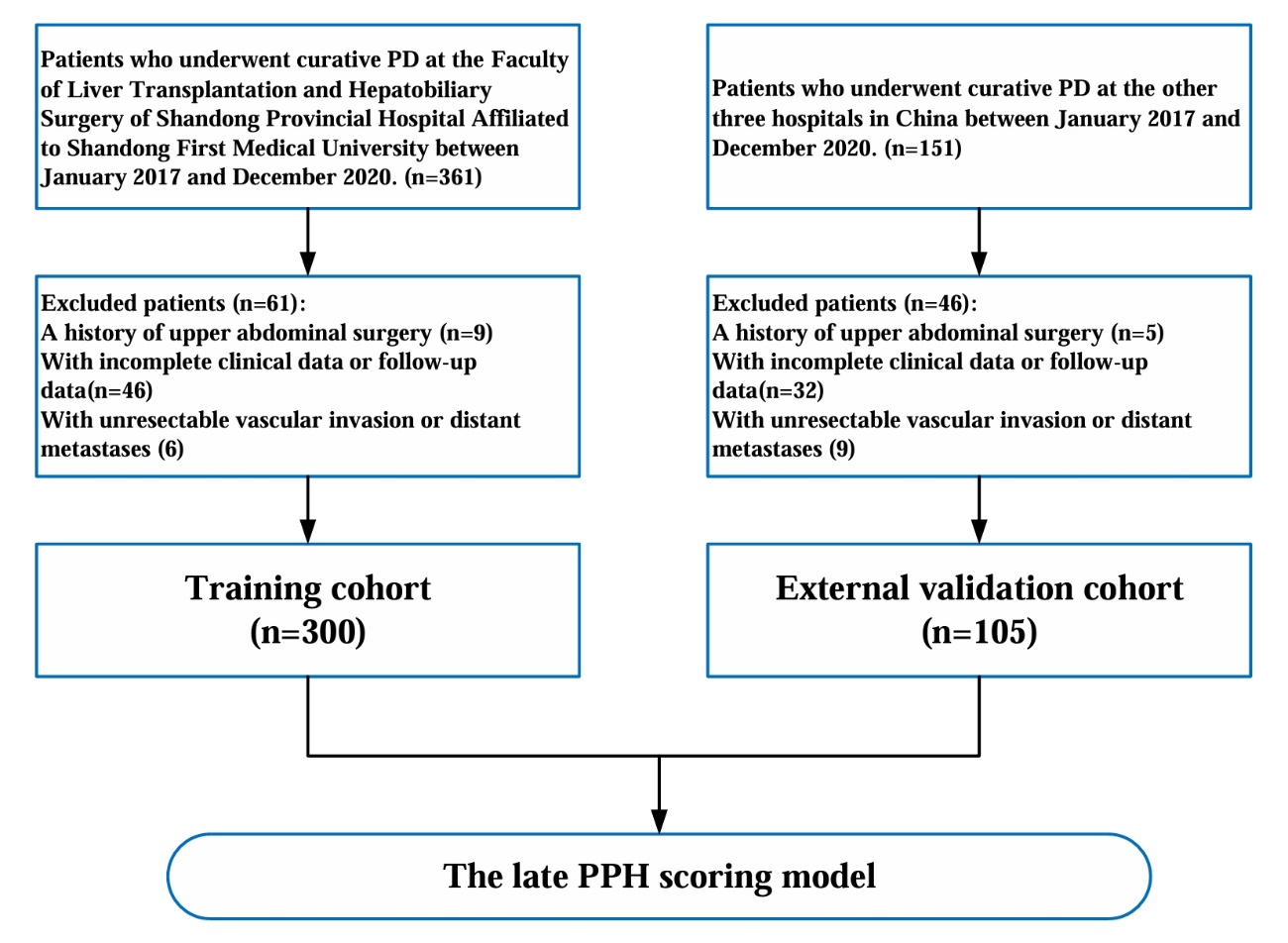


# Supplementary Table 1. Univariate logistic analysis for Late PPH after pancreaticoduodenectomy

| **Characteristics** | **B** | **SE** | **OR (95% CI)** | ***P* value** |
| --- | --- | --- | --- | --- |
| Age (>65 years vs. ≤65 years) | 0.885 | 0.437 | 2.423 (1.029-5.707) | **0.043** |
| Gender (male vs. female) | 0.514 | 0.469 | 1.671 (0.666-4.193) | 0.274 |
| BMI (>25.8 kg/m^2^ vs. ≤25.8 kg/m^2^) | 1.000 | 0.454 | 2.717 (1.117-6.612) | **0.028** |
| History of HBP (yes vs. no) | 0.592 | 0.478 | 1.807 (0.708-4.509) | 0.216 |
| ASA stage (≥III vs.≤II) | 0.767 | 0.505 | 2.154 (0.800-5.799) | 0.129 |
| History of smoking (yes vs. no) | 0.423 | 0.499 | 1.527 (0.574-4.062) | 0.396 |
| History of alcohol use (yes vs. no) | 0.505 | 0.656 | 1.657 (0.458-5.996) | 0.442 |
| Preoperative biliary drainage (yes vs. no) | -0.208 | 0.570 | 0.813 (0.266-2.483) | 0.716 |
| Preoperative TBIL (>2.0 mg/dL vs. ≤2.0 mg/dL) | 1.245 | 0.520 | 3.472 (1.254-9.614) | **0.017** |
| Preoperative ALB (<35 g/L vs. ≥35 g/L) | -0.910 | 0.756 | 0.403 (0.092-1.770) | 0.228 |
| Preoperative BG (>6.1 mmol/L vs. ≤6.1 mmol/L) | -0.069 | 0.494 | 0.933 (0.355-2.456) | 0.889 |
| Preoperative PLT (<300*10^9^/L vs. ≥300*10^9^/L) | -0.569 | 0.478 | 0.566 (0.222-1.444) | 0.234 |
| Preoperative PT (>14.4 S vs. ≤14.4 S) | 1.447 | 0.564 | 4.248 (1.406-12.835) | **0.010** |
| Preoperative APTT (>33.2 S vs. ≤33.2 S) | 0.993 | 0.755 | 2.543 (0.578-11.176) | 0.217 |
| Preoperative INR (>1.08 vs. ≤1.08) | 0.701 | 0.450 | 2.015 (0.835-4.865) | 0.119 |
| Preoperative FIB (<4g/L vs.≥4g/L) | -0.590 | 0.438 | 0.554 (0.235-1.307) | 0.178 |
| Surgical approach (MIPD vs. Open) | -0.786 | 0.436 | 0.456 (0.194-1.072) | 0.072 |
| Operative time (>360 min vs. ≤360 min) | 0.790 | 0.540 | 2.204 (0.765-6.355) | 0.143 |
| Estimated blood loss (>200 ml vs. ≤200 ml) | 0.344 | 0.458 | 1.411 (0.575-3.462) | 0.453 |
| Largest tumor diameter (>2.5 cm vs. ≤2.5 cm) | -0.514 | 0.469 | 0.598 (0.239-1.501) | 0.274 |
| Peripheral tissue invasion (yes vs. no) | 0.432 | 0.522 | 1.540 (0.553-4.287) | 0.408 |
| Neoadjuvant chemotherapy (yes vs. no) | 0.743 | 0.797 | 2.103 (0.441-10.024) | 0.351 |
| Vascular resection (yes vs. no) | -0.231 | 1.056 | 0.794 (0.100-6.295) | 0.827 |
| Resection margin (R0 vs. non-R0) | -0.370 | 0.653 | 0.691 (0.192-2.481) | 0.570 |
| Prophylactic anticoagulation (yes vs. no) | 0.194 | 1.072 | 1.214 (0.148-9.922) | 0.857 |
| Pathology (malignant vs. benign) | -0.257 | 0.777 | 0.773 (0.169-3.548) | 0.741 |
| Tumor location |  |  |  |  |
| Pancreas |  |  | ref |  |
| Bile duct | 0.782 | 0.478 | 2.187 (0.857-5.582) | 0.102 |
| Duodenum | -0.475 | 0.693 | 0.622 (0.160-2.418) | 0.493 |
| Tumor differentiation |  |  |  |  |
| Benign and well |  |  | ref |  |
| Moderate | 0.615 | 0.596 | 1.850 (0.575-5.949) | 0.302 |
| Poor | 0.445 | 0.647 | 1.560 (0.439-5.544) | 0.492 |
| CR-POPF (yes vs. no) | 2.618 | 0.472 | 13.704 (5.436-34.546) | **<0.001** |
| Bile leak (yes vs. no) | -0.483 | 1.050 | 0.617 (0.079-4.830) | 0.646 |
| Gastrointestinal fistula (yes vs. no) | 1.132 | 1.140 | 3.102 (0.332-28.965) | 0.321 |

Bold text hinted that these variables were statistically significant in univariate analysis; Abbreviation: OR: Odds Ratio. B: Coefficient; SE: Standard error; CI: Confidence interval. BMI: Body Mass Index; HBP: High blood pressure; ASA: American Society of Anesthesiologists; Hb: Hemoglobin; WBC: White Blood Cell; PLT: Platelet; PT: Prothrombin time; APTT: Activated partial thromboplastin time; INR: International Normalized Ratio; FIB: Fibrinogen; BG: Blood glucose; ALB: Albumin; TBIL: Total bilirubin; MIPD, minimally invasive pancreatoduodenectomy; CR-POPF: Clinically relevant postoperative pancreatic fistula; PPH: Post pancreatectomy hemorrhage

# Supplementary Table 2. Late PPH incidence of different score in the entire cohort (n=405)

| **Late PPH model score** | **Number of people** | **Late PPH, Yes/No** | **Late PPH rate** |
| --- | --- | --- | --- |
| **0** | 136 | 1/135 | 0.7% |
| **1** | 170 | 6/164 | 3.5% |
| **2** | 27 | 2/25 | 7.4% |
| **3** | 31 | 4/27 | 12.9% |
| **4** | 28 | 11/17 | 39.3% |
| **5** | 9 | 4/5 | 44.4% |
| **6** | 4 | 3/1 | 75.0% |

**Abbreviations:** PPH: Post pancreatectomy hemorrhage.

# Supplementary Table 3. Late PPH incidence of different risk grade

| **Cohort** | **Risk grade** | **Late PPH** | **Non-Late PPH** | ***P* value** |
| --- | --- | --- | --- | --- |
| **Training cohort**  **(n=300)** | High Risk (score>2) (n=55) | 16 (29.1%) | 39 (70.9%) | <0.001 |
|  | Low Risk (score≤2) (n=245) | 7 (2.9%) | 238 (97.1%) |  |
| **Validation cohort**  **(n=105)** | High Risk (score>2) (n=17) | 6 (35.3%) | 11 (64.7%) | <0.001 |
|  | Low Risk (score≤2) (n=88) | 2 (2.3%) | 86 (97.7%) |  |
| **Entire cohort**  **(n=405)** | High Risk (score>2) (n=72) | 22 (30.6%) | 50 (69.4%) | <0.001 |
|  | Low Risk (score≤2) (n=333) | 9 (2.7%) | 324 (97.3%) |  |

**Abbreviations:** PPH: Post pancreatectomy hemorrhage.

# Supplementary Table 4. Multicollinearity Assessment (VIF and Tolerance)

| **Variable** | **Variance Inflation Factor (VIF)** | **Tolerance** |
| --- | --- | --- |
| BMI (>25.8 vs. ≤25.8 kg/m²) | 1.43 | 0.70 |
| Preoperative TBIL (>2.0 vs. ≤2.0 mg/dL) | 1.62 | 0.62 |
| Preoperative PT (>14.4 vs. ≤14.4 s) | 1.57 | 0.64 |
| CR-POPF (yes vs. no) | 1.31 | 0.76 |

**Abbreviations:** BMI: Body Mass Index; PT: Prothrombin time; TBIL: Total bilirubin; CR-POPF: Clinically relevant postoperative pancreatic fistula.

.
